# High prevalence of hepatitis delta virus among people with hepatitis B virus and HIV coinfection in Botswana

**DOI:** 10.1016/j.jiph.2023.08.011

**Published:** 2023-11

**Authors:** Kabo Baruti, Bonolo B. Phinius, Basetsana Phakedi, Gorata Mpebe, Wonderful Choga, Lynnette Bhebhe, Graceful Mulenga, Natasha O. Moraka, Tsholofelo Ratsoma, Molly Pretorius-Holme, Joseph Makhema, Roger Shapiro, Shahin Lockman, Sikhulile Moyo, Mosimanegape Jongman, Motswedi Anderson, Simani Gaseitsiwe

**Affiliations:** a Research laboratory, Botswana Harvard AIDS Institute Partnership, Gaborone, Botswana; b Department of Biological Sciences, Faculty of Science, University of Botswana, Gaborone, Botswana; c School of Allied Health Professions, Faculty of Health Sciences, University of Botswana, Gaborone, Botswana; d Department of Immunology and Infectious Diseases, Harvard T.H. Chan School of Public Health, Boston MA, United States

**Keywords:** Prevalence, Hepatitis delta virus, People with HIV, Botswana

## Abstract

**Background:**

Approximately 15–20 million people worldwide are infected with hepatitis delta virus (HDV), which is approximately 5 % of people with chronic hepatitis B virus (HBV). Sub-Saharan Africa has high HDV prevalence, leading to worse clinical outcomes among people who are HIV/HBV/HDV tri-infected. There are limited data on HDV prevalence among people with HIV (PWH) who are HBV-infected and uninfected in Botswana. We, therefore, determined HDV prevalence among PWH in Botswana.

**Methods:**

This was a retrospective cross-sectional study utilizing archived plasma samples from PWH with results for HBV markers such as hepatitis B surface antigen (HBsAg), hepatitis B core antibody (anti-HBc), immunoglobulin M antibody to hepatitis B core antigen (IgM anti-HBc) and hepatitis B e antigen (HBeAg). Samples were categorized according to their HBsAg status and screened for anti-HDV antibodies. Total nucleic acid was extracted from samples with a single positive anti-HDV result, and HDV ribonucleic acid (RNA) load was quantified using the Altona Diagnostic RealStar® HDV RT-PCR kit. Statistical analysis was performed using STATA version 14.0 where p-values < 0.05 were considered statistically significant.

**Results:**

The study cohort (n = 478) included both HBsAg positive (44 %) and negative (56 %) participants, with a median age of 42 [IQR; 41–43]. Anti-HDV prevalence of (15/211) [7.1 %, 95 % CI: 4.4 – 11.4] was recorded among HBsAg positive participants, all of whom were IgM anti-HBc negative, while 5/6 participants were HBeAg negative. HDV RNA load was detected in 11/12 (92 %) anti-HDV-positive participants. No HDV prevalence was recorded among participants who were HBsAg negative, therefore, the overall HDV prevalence was (15/478) [3.1 %, 95 % CI: 1.9 – 5.1]. HIV viral load suppression was statistically insignificant, irrespective of HDV status.

**Conclusions:**

We report high HDV prevalence among HBsAg-positive PWH in Botswana. Most HDV-positive participants had active HDV infection, therefore, we recommend HDV screening in this cohort to guide their clinical care.

## Introduction

Hepatitis delta virus (HDV) is a defective, obligate satellite virus of hepatitis B virus (HBV), which requires hepatitis B surface antigen (HBsAg) proteins from its helper virus for viral particle formation [1]. HBV/HDV coinfection or superinfection in chronically infected HBV patients accelerates and worsens the progression of liver disease and triples the risk of developing hepatocellular carcinoma (HCC) [2]. Chronic hepatitis D (CHD) is the most severe form of viral hepatitis infection [3].

It is estimated that 15–20 million of the 296 million people with chronic HBV are infected with HDV [2], which translates to approximately 4.5 % prevalence [4]. The highest prevalence is reported in regions endemic for HBV such as Eastern and Mediterranean Europe, sub-Saharan Africa (SSA) as well as Central, Eastern and Northern Asia [5], [6]. In SSA, the prevalence of anti-HDV antibodies seems even higher among human immunodeficiency virus (HIV)/HBV-coinfected patients, reaching 25 % in Guinea Bissau [7]. Concomitant HIV/HBV/HDV infection has been associated with worse clinical outcomes than HBV/HDV infection, resulting in a higher incidence of hepatic flares and decompensation as well as increased mortality [8]. Despite this, a study reported declining and ultimately undetectable HDV ribonucleic acid (RNA) levels among HIV/HBV/HDV tri-infected patients who were receiving tenofovir and interferon-based therapy [9]. However, the long-term implications of these findings remain unknown.

Although HDV infection is thought to influence HBV activities, the exact mechanisms of interaction between these viruses are yet to be determined [10]. However, retrospective studies have found the presence of HDV infection to reduce HBV deoxyribonucleic acid (DNA) levels in patients’ blood. A longitudinal study reported high levels of HDV RNA and low levels of HBV DNA among 54 % of the participants, low levels of HDV RNA and high levels of HBV DNA (30 % of patients), and equal levels of HDV RNA and HBV DNA in 15 % of patients [11]. These findings may suggest subtle HDV dominance over HBV among people with HBV/HDV infection.

Interestingly, an in-vivo study reported HDV infection without HBV as the helper virus [12]. It has been hypothesized that enveloped viruses such as the West Nile virus, Dengue virus and vesiculovirus used in this study might have the ability to act as helper viruses for HDV, providing it with envelope proteins needed during replication. A more recent study detected anti-HDV antibodies in the absence of HBsAg and other HBV markers such as HBV DNA and hepatitis B core antibodies (anti-HBc) [13]. These two studies indicate that the true HDV burden may be underestimated as studies have always focused on people with positive HBsAg results.

In Botswana, two previous studies reported high anti-HDV prevalence among HIV/HBV co-infected patients (75 %), albeit with a small sample size (n = 4), and people presenting hepatitis symptoms (69 %), while the third study reported 4.6 % prevalence among liver disease patients [14], [15], [16]. Therefore, there are limited data on HDV prevalence among people with HIV (PWH) in Botswana. Estimating HDV prevalence among PWH is critical in guiding clinical care, formulating policies and informing effective public health interventions. This is because undiagnosed HDV may be assumed to be HBV infection and treated with drugs which have anti-HBV activity, but these are less effective in clearing HDV RNA. We therefore sought to determine HDV prevalence among HBsAg positive and negative PWH who were enrolled in a large HIV prevention trial cohort conducted between 2013 and 2018 in Botswana.

## Materials and methods

### Study participants

This was a retrospective cross-sectional study utilizing 478 archived residual plasma samples from the “*Ya Tsie”* also referred to as the Botswana Combination Prevention Project (BCPP) (2013–2018). The BCPP study was a pair-matched cluster-randomized trial which enrolled a total of 12 610 participants, 3596 of whom were PWH with an age range (16–64 years) from 30 communities across Botswana [17]. Half of the communities were randomly assigned to receive HIV prevention and treatment interventions, including enhanced HIV testing, earlier antiretroviral therapy (ART), and strengthened male circumcision services, while 15 received standard of care. Residual samples from the parent study had been stored at −80 °C in an ultralow temperature freezer. Participants chosen for this study were PWH who had previously been screened for HIV as described in the parent study [17]. Briefly, information about HIV testing history (including date, location, and result) was collected at each visit and participants without documented HIV-positive status were screened using parallel HIV rapid tests (KHB, Shanghai Kehua Bio-Engineering, Shanghai, China, and Unigold, Trinity Biotech, Bray, Wicklow, Ireland). HIV-1 RNA was tested in all people living with HIV at the baseline and final visits, irrespective of ART use (Abbott RealTime HIV-1 Assay, Wiesbaden, Germany). Viral suppression was defined as HIV-1 RNA less than or equal to 400 copies per mL. Point-of-care CD4 cell count (Pima, Alere, Waltham, MA, USA) was obtained on people living with HIV who were not on ART. Documentation of ART (eg, prescriptions or pills, clinical notes) was required for classifying a participant as on ART.

These participants were further screened for HBV serological markers as previously described [18]. Briefly, plasma samples were screened for HBsAg (Murex Version 2, Diasorin, Dartford, United Kingdom) and anti-HBc using the Monolisa anti-HBc PLUS enzyme-linked immunosorbent assay kit (Bio-Rad, Marnes-la-Coquette, France). HBsAg positive samples were further screened for hepatitis B e antigen (HBeAg) and anti-HBc immunoglobulin M (IgM anti-HBc) using the Monolisa HBe Ag/Ab and Monolisa anti-HBc Plus 1 Plaque (Bio-Rad, Marnes-la-Coquette, France), respectively. HBV DNA was quantified in HBsAg positive participants using the COBAS AmpliPrep/COBAS TaqMan HBV Test version 2.0 (Roche Diagnostics, Mannheim, Germany) following the manufacturer’s instructions with a broad linear range from 20 to 1.7 × 108 IU/ mL.

Demographics data such as age, marital status, education and gender as well as clinical characteristics such as ART status and regimen were retrieved from the participants data sheet. Their samples were categorized according to their HBsAg status and screened for HDV using one time point.

### Anti-HDV testing

Samples were screened for anti-HDV at least once using the General Biologicals HDV Ab kit (General Biologicals Corporation, Taiwan, China), as per the manufacturer’s instructions to determine HDV prevalence.

### Total nucleic acid extraction

Total nucleic acid (TNA) was extracted from samples that tested positive for anti-HDV at least once. This was performed using the DaAn Gene nucleic acid extraction kit (DaAn Gene Co, Ltd., China) which required a sample volume of 200 µl as per manufacturer's instructions. A TNA volume of 50 µl was eluted and stored in small aliquots at −20 °C.

### HDV RNA load quantification

HDV viral RNA was determined with the Altona Diagnostic RealStar® HDV RT-PCR 1.0 detection kit, with slight modifications in the manufacturer’s instructions. The master mix was prepared by adding Master A and B which contain all components (PCR buffer, reverse transcriptase, DNA polymerase, magnesium salt, primers and probes) with the internal control to make a total volume of 27.5 µl. Then 12.5 µl from the master mix was added to the sample (12.5 µl) to make a final reaction volume of 25 µl which was then amplified following the manufacturer’s instructions. Quantification standards with different concentrations (1000 IU/µl, 100 IU/µl, 10 IU/µl and 1 IU/µl) were used to draw a standard curve to calculate the HDV RNA load as per the manufacturer’s instructions.

### Statistical analysis

STATA v.14.0 (Stata Corporation, College Station, TX, USA) was used for data analysis where p-values< 0.05 were considered statistically significant. HDV prevalence was calculated using Wilson 95 % confidence intervals. A statistical comparison of demographics between HDV positive and HDV negative participants was performed using the Pearson Chi-square or Fisher’s exact method and the Mann-Whitney U test where applicable.

## Results

**Fig. 1:**
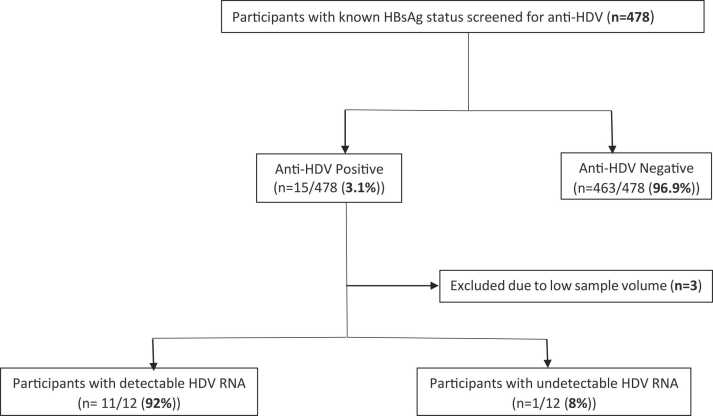
Hepatitis D virus screening algorithm flow chart for the study participants.

Plasma samples from a total of 478 participants with known HBsAg status, majority of whom were females (70.6 %), were further screened for HDV. The study cohort included HBsAg positive (211/478) and HBsAg negative (267/478) participants and they were screened following the algorithm in Fig. 1.

An anti-HDV prevalence of (15/211) [7.1 %, 95 % CI: 4.4 – 11.4] was recorded among HBsAg positive participants, all of whom were IgM anti-HBc negative (Table 1). No anti-HDV prevalence was recorded among participants who were HBsAg negative, therefore, the overall anti-HDV prevalence was (15/478) [3.1 %, 95 % CI: 1.9 – 5.1].

**Table 1:** Demographics and clinical characteristics.

**Characteristic**	**HDV positive**	**HDV negative**	**p-value**
**Male n (%)**	6 (40)	137 (30)	0.39
**Median Age [IQR]**	44 [34–56]	42 [35–49]	0.40
**HIV viral load** (n = 417**)** Suppressed Not suppressed	6 (86) 1 (14)	330 (78) 92 (22)	0.63
**ART Status** (n = 476) On ART Naive	15 (100) 0	381 (83) 80 (17)	0.08
**ART regimen** (n = 281) Non-3TC, non-TDF containing regimen TDF containing regimen 3TC containing regimen	0 7 (50) 7 (50)	11 (4) 141 (53) 115 (43)	0.69
**ARV duration in months median (IQR) years**	7.8 (3.8–9.6)	6.6 (3.3–9.5)	0.46
**HBV viral load** (n = 126) Target Not Detected < 20, 000 IU/mL ≥ 20, 000 IU/mL	2 (33) 3 (50) 1 (17)	30 (25) 76 (63) 14 (12)	0.80
**HBeAg positivity, n (%)**	1 (17)	16 (11)	0.52
**Anti-HBc Positivity, n (%)**	7 (100)	281 (68)	0.07
**IgM anti-HBc Positivity, n (%)**	0	11 (6)	1.0
**Marital status, n (%)** (n = 478) Single or never Married Married, divorced, separated or widowed	11 (73) 4 (27)	351 (76) 112 (24)	0.83
**Education, n (%)** (n = 475) Non-formal and Primary Junior Secondary education or higher	11 (73) 4 (27)	205 (45) 255 (55)	0.03

HDV- Hepatitis D virus, HIV- human immunodeficiency virus, IQR- interquartile range, HBV-hepatitis B virus, HBeAg-hepatitis B e antigen, IgM anti-HBc- Immunoglobulin M antibody to hepatitis B core antigen, Anti-HBc-hepatitis B core antibody, ARV-antiretrovirals

Additionally, there were no statistically significant differences between HDV-positive and HDV-negative participants in terms of marital status and positivity for HBV markers. Interestingly, none of the HDV-positive participants were IgM anti-HBc positive, while all the seven (7) participants who had anti-HBc data tested positive.

Table 2 shows virologic information about HDV positive participants. HDV RNA was detected in 11/12 (92 %) confirmed anti-HDV positive participants who had a detectable HBV viral load despite receiving a combined antiretroviral therapy (cART) with anti-HBV activity (Table 2).

**Table 2:** Characteristics of HDV positive participants.

ID	Gender	Age	Community	HBsAg	HDV RNA load (IU/mL)	HBV DNA load (IU/mL)	HIV viral load (IU/mL)	Anti-HBc	ARV regimen	ARV duration (Years)	IgM anti HBc	HBeAg
1	F	36	Gumare	POS	417496	41	N/A	N/A	3TC + NVP + ZDV	7.8	NEG	N/A
2	M	45	Gumare	POS	172871	<20	N/A	N/A	FTC + LPV + TDF	3.8	NEG	N/A
3	F	40	Gumare	POS	2569776	481	N/A	N/A	EFV + FTC + TDF	2.1	NEG	N/A
4	M	51	Gumare	POS	13127	TND	N/A	N/A	3TC + EFV + ZDV	12.4	NEG	N/A
5	M	54	Gweta	POS	16	2086	40	POS	EFV + FTC + TDF	8.4	NEG	NEG
6	M	57	Rakops	POS	148078	36	40	POS	N/A	9.6	NEG	NEG
7	M	58	Gumare	POS	93696	<20	40	POS	3TC + EFV + ZDV	9.1	NEG	NEG
8	F	38	Gumare	POS	2090	#N/A	40	POS	3TC + NVP + ZDV	11.6	NEG	NEG
9	F	32	Sebina	POS	1010	#N/A	40	POS	3TC + NVP + ZDV	6.4	NEG	NEG
10	F	34	Shakawe	POS	TND	247	N/A	N/A	EFV + FTC + TDF	4.1	NEG	N/A
11	F	35	Rakops	POS	72	>170000000	21574	POS	FTC + TDF	6.7	NEG	POS
12	F	56	Nkange	POS	61	TND	40	POS	3TC + EFV + ZDV	10.7	NEG	N/A
13	M	60	Gumare	POS	N/A	254	N/A	N/A	EFV + FTC + TDF	3.5	NEG	N/A
14	F	33	Shakawe	POS	N/A	1399	N/A	N/A	3TC + NVP + ZDV	9.4	NEG	N/A
15	F	44	Shakawe	POS	N/A	409	N/A	N/A	EFV + FTC + TDF	3.7	NEG	N/A

M-Male, F-Female, TND-Target Not Detected, POS-Positive, NEG-Negative, N/A-Information not available, HBsAg-hepatitis B surface antigen, HDV-hepatitis delta virus, HBV- hepatitis B virus, HIV-human immunodeficiency virus, anti-HBc-core antibody to the core antigen, ARV-antiretroviral, IgM anti-HBc- Immunoglobulin M antibody to hepatitis B core antigen, HBeAg- hepatitis B e antigen, 3TC-Lamivudine, NVP-Nevirapine, ZDV-Zidovudine, FTC-Emtricitabine, LPV-Lopinavir, TDF-Tenofovir, EFV-Efivarenz, DNA- Deoxyribonucleic acid, RNA- Ribonucleic acid

All HDV-positive participants were HBsAg positive and their HBV DNA and HDV RNA loads were inversely proportional. High HDV RNA load and low HBV DNA load were found in 60 % of the participants, low HDV RNA load and high HBV DNA load were found in 30 % of the participants while 10 % had low HDV RNA load and undetectable HBV DNA load.

## Discussion

We determined the HDV prevalence among PWH in Botswana. This study is important because previous studies have reported a moderate to high prevalence of HBV among PWH in Botswana [19], [20], [21], [22], [23]. People with HBV infection are at risk of HDV infection as HBV provides HDV with envelope proteins required for its replication. Furthermore, it is imperative to determine the presence of HDV among PWH due to shared routes of transmission and reported worse clinical outcomes associated with HIV/HBV/HDV tri-infection. We report 7.1 % and no anti-HDV prevalence among PWH who were HBsAg positive and negative, respectively, and 3.1 % overall anti-HDV prevalence in both cohorts.

The reported HDV prevalence among HBsAg positive participants (7.1 %) in our study is higher than that previously reported in Botswana and the globe, which stand at 2.1–5.0 % and 5 %, respectively [4]. A recent study from a cohort similar to ours revealed high HDV prevalence (75 %), which may have been influenced by the small sample size of only four participants [16]. Interestingly, previous studies in Guinea-Bissau and Cameroon have also reported high HDV prevalence of 25 % and 43.3 %, respectively, in a cohort of HIV/HBV-infected individuals [7], [24]. The study in Cameroon, which is endemic to HBV, also revealed a high HDV prevalence (56.7 %) among HBsAg-positive people who were without HIV [24]. However, HDV detection among people without HIV in the Cameroon study may suggest the need to screen for HDV among HBsAg-positive people regardless of HIV status. All this data reveals that HDV prevalence in Africa may be higher than estimated by the World Health Organization (WHO).

HDV infection was not detected among HBsAg-negative participants in our study. Two recent studies detected HDV infection accompanied by the absence of HBV infection despite screening for serological and molecular HBV markers, while another one revealed successful propagation of HDV using enveloped viruses such as vesiculovirus, flavivirus and hepacivirus [12], [13]. Analysis of these two studies seemed to suggest that HDV disease pathogenesis may be evolving, leading to less dependence on HBV for replication. However, our results did not support their findings, but instead revealed a high dependence on HBV, as evidenced by no HDV detection among HBsAg-negative people.

Despite findings from our study and previous literature suggesting that HDV leads to a higher HIV viral load, there was no statistically significant difference between HDV positive and HDV negative participants with respect to HIV viral load suppression in our study. We did not observe any statistically significant difference between HDV positive and negative participants in terms of clinical characteristics and demographics (except for education). Very few studies have reported on the effect of HDV on HBV serological markers hence it is worth noting that none of the HDV positive participants were IgM positive, while all of those with anti-HBc data (n = 7) were positive, despite the data not being statistically significant. According to our findings, there were more female participants with HBV/HDV coinfection (60 %) than males, albeit with no statistically significant difference. These findings are similar to what was reported by a previous study which also reported higher HDV prevalence among female liver disease patients in Botswana, but without statistical significance [15]. These two findings suggest that gender might not be a risk factor for HDV infection, which is contradictory to other studies which have reported higher HDV prevalence in males [25], [26], [27]. The exact reason(s) for the reported high HDV prevalence in either gender are yet to be elucidated.

HDV RNA load was detected in 92 % of anti-HDV positive participants with enough sample volume, suggesting that they had an active HDV infection. Only one anti-HDV positive participant had an undetectable HDV RNA load, which might suggest that they had resolved HDV infection. Consistent with the available literature, which reported subtle HDV dominance over HBV [11], HDV was the predominant virus in 60 % of participants with HDV RNA and HBV DNA load data. HDV and HBV are reported to compete for the HBsAg during virus replication, so the HBsAg becomes insufficient to support the existence of both viruses. HDV has developed proteins which have the capability to inhibit HBV activity by trans-repressing the HBV enhancer and by trans-activating the IFNa-inducible MxA-gene [11]. This, coupled with the fact that all HDV infected participants were receiving anti-HBV active ART regimen for a median duration of 7.8 years (3.8–9.6), may partly explain why HDV was the predominant virus.

Study limitations include missing data such as HIV viral load and liver enzyme levels which could have allowed us to determine the relationship between HDV and HIV viral loads and assess liver damage in HIV/HBV/HDV tri-infected participants. The lack of HIV negative individuals is a limitation because it would have helped determine whether HIV status plays a significant role in HDV prevalence.

In conclusion, we report relatively high HDV prevalence among HIV/HBsAg positive participants, most of whom had active HDV infection due to HDV RNA load detection. Our study also revealed no anti-HDV prevalence among HBsAg negative individuals, therefore introduction of HDV screening in HBV/HIV infected individuals is recommended. Future studies should focus on determining the circulating HDV clades and their impact on clinical outcomes in this cohort and compare with HIV-negative individuals.

## Ethical considerations

The study participants signed informed written consent in the parent study for participation in the BCPP study, and for storage and usage of residual samples in future studies. The study received ethical clearance from the University of Botswana (UB) ethics review committee and was granted a research permit (HPRD: 6/14/1) from the Health Research Development Committee (HRDC) of the Botswana Ministry of Health (MoH).

## Funding

This work was supported by the 10.13039/100004440Wellcome Trust (grant number 218770/Z/19/Z). WTC, SM and SG are partly supported through the Sub-Saharan African Network for TB/HIV Research Excellence (SANTHE 2.0) from the 10.13039/100000865Bill & Melinda Gates Foundation (INV-033558). BBP and SG are supported by the 10.13039/100000002National Institutes of Health (NIH) Common Fund, award number U41HG006941 (H3ABioNet). H3ABioNet is an initiative of the Human Health and Heredity in Africa Consortium (H3Africa) program of the African Academy of Science. BBP was partially supported by Trials of Excellence in Southern Africa (TESAIII), which is part of the EDCTP2 program supported by the 10.13039/501100000780European Union (grant number CSA2020NoE-3104 TESAIII). SL, RS and SM received support from the 10.13039/100000002NIH (awards numbers K24 AI131928, K24 AI131924 and 1K43TW012350-01), respectively). SG and SM were supported by the Fogarty International Center at the US National Institutes of Health Awards, D43 TW009610 and 1K43TW012350-01, respectively. The views expressed in this publication are those of the authors and not necessarily those of European Union, EDCTP, NIH, Bill & Melinda Gates Foundation, Africa Academy of Sciences or UK government. The funders had no role in the study design, data collection and decision to publish, or in the preparation of the manuscript.

## CRediT authorship contribution statement

Kabo Baruti, Simani Gaseitsiwe and Motswedi Anderson designed the study and methodology. Simani Gaseitsiwe, Motswedi Anderson, Bonolo B. Phinius, Roger Shapiro, Shahin Lockman, Molly Pretorius-Holme and Joseph Makhema provided resources for the study. Kabo Baruti, Sikhulile Moyo, Bonolo B. Phinius, Natasha O, Moraka and Graceful Mulenga performed the statistical analysis. All the authors were part of the manuscript writing and editing team while Simani Gaseitsiwe, Sikhulile Moyo, Motswedi Anderson and Mosimanegape Jongman provided supervision. All authors have read and given final approval for the manuscript version to be published.

## Declaration of Competing Interest

We have no conflict of interest to declare.
